# Inclusion Complexes of Melphalan with Gemini-Conjugated β-Cyclodextrin: Physicochemical Properties and Chemotherapeutic Efficacy in In-Vitro Tumor Models

**DOI:** 10.3390/pharmaceutics11090427

**Published:** 2019-08-22

**Authors:** Waleed Mohammed-Saeid, Abdalla H Karoyo, Ronald E Verrall, Lee D Wilson, Ildiko Badea

**Affiliations:** 1 Drug Discovery and Development Research Group, College of Pharmacy and Nutrition, University of Saskatchewan, 107 Wiggins Rd, Saskatoon, SK S7N 5E5, Canada; 2 College of Pharmacy, Taibah University, Medina 42353, Saudi Arabia; 3 Department of Chemistry, University of Saskatchewan, 110 Science Place, Saskatoon, SK S7N 5C9, Canada

**Keywords:** cationic gemini surfactant, melphalan, inclusion complex, ROESY NMR spectroscopy, 3D spheroid, drug-resistant melanoma

## Abstract

β-cyclodextrin (βCD) has been widely explored as an excipient for pharmaceuticals and nutraceuticals as it forms stable host–guest inclusion complexes and enhances the solubility of poorly soluble active agents. To enhance intracellular drug delivery, βCD was chemically conjugated to an 18-carbon chain cationic gemini surfactant which undergoes self-assembly to form nanoscale complexes. The novel gemini surfactant-modified βCD carrier host (hereafter referred to as 18:1βCDg) was designed to combine the solubilization and encapsulation capacity of the βCD macrocycle and the cell-penetrating ability of the gemini surfactant conjugate. Melphalan (Mel), a chemotherapeutic agent for melanoma, was selected as a model for a poorly soluble drug. Characterization of the 18:1βCDg-Mel host–guest complex was carried out using 1D/2D ^1^H NMR spectroscopy and dynamic light scattering (DLS). The 1D/2D NMR spectral results indicated the formation of stable and well-defined 18:1βCDg-Mel inclusion complexes at the 2:1 host–guest mole ratio; whereas, host–drug interaction was attenuated at greater 18:1βCDg mole ratio due to hydrophobic aggregation that accounts for the reduced Mel solubility. The in vitro evaluations were performed using monolayer, 3D spheroid, and Mel-resistant melanoma cell lines. The 18:1βCDg-Mel complex showed significant enhancement in the chemotherapeutic efficacy of Mel with 2–3-fold decrease in Mel half maximal inhibitory concentration (IC_50_) values. The findings demonstrate the potential applicability of the 18:1βCDg delivery system as a safe and efficient carrier for a poorly soluble chemotherapeutic in melanoma therapy.

## 1. Introduction

Melanoma, the malignant cancer of melanocytes, is the most aggressive form of skin cancer which causes the most skin-cancer related deaths [1]. According to the World Health Organization (WHO), over 132,000 new cases of melanoma are diagnosed annually [2]. In its early stages, melanoma can be treated by surgical incision with high survival rate. In the stage of in-transit metastases, in which the metastases are >2 cm from the primary lesion but within the nodal basin, the response to local and systemic therapeutic options is moderate with 5-year survival of 32.8% [3,4]. However, advanced metastatic melanoma shows limited response to current therapeutic options with very low survival rate of less than 5% over 5 years [5]. Systemic chemotherapy is the first-line option for most patients with metastatic melanoma.

**Figure 1:**
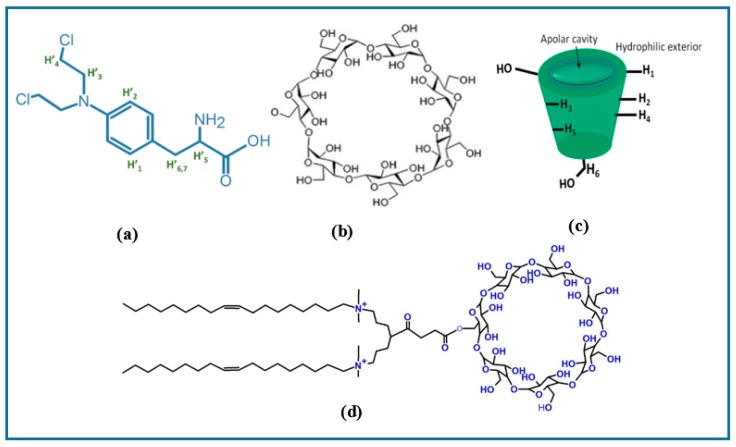
Chemical structures of (**a**) Melphalan (Mel), (**b**) β-Cyclodextrin (βCD) macrocycle, (**c**) βCD toroidal structure showing cavity and external protons, and (**d**) 18:1βCDg host (carrier).

Melphalan (Mel) (Figure 1a) is used regionally as an adjunctive therapy for in-transit metastatic melanoma [6]. The lipophilic nature of Mel requires the use of a co-solvent (e.g., propylene glycol) for parenteral administration. Propylene glycol is known to cause toxicity that includes nephrotoxicity, cardiac arrhythmia, and metabolic acidosis [7]. As a result, the use of Mel in melanoma therapy is limited to isolated limb perfusion/infusion which is an invasive method that requires special medical care [6]. Therefore, attempts to improve the solubility and stability of Mel were conducted by either chemical modification of the molecule or by engineering novel drug delivery systems, such as nano-systems [8,9,10,11]. A nanoscale drug delivery system has several advantages, it can: (1) improve solubility of poorly soluble drugs, (2) enhance chemical/biological stability, (3) improve pharmacokinetic profile and biodistribution, (4) increase tumor-specific uptake (passive targeting), (5) minimize drug resistance, (6) achieve drug controlled release, and (7) afford delivery of multiple drug components [12,13]. Our strategy is to create delivery systems that could improve the therapeutic use of Mel addressing both issues of solubility and biological activity at the same time. Cyclodextrins (CDs) form stable host–guest complexes with a variety of organic and inorganic molecules and have been widely employed as versatile carriers for poorly soluble drugs [14]. CDs (Figure 1b,c) are naturally occurring cyclic oligosaccharides consisting of 6 (αCD), 7 (βCD), or 8 (γCD) α-d-glucopyranose units linked by (α-1,4) glycosidic bonds [15]. CDs form a truncated cone with a toroidal structure (Figure 1c) in which the hydroxyl groups of glucopyranose units reside at the narrow (primary) and wide (secondary) tori of the CD annular structure. The CD macrocycle has a hydrophilic outer surface and lipophilic inner cavity that is capable of forming noncovalent inclusion complexes with a large variety of guest molecules [16]. Capitalizing on the ability of the CDs to form host–guest inclusion complexes, such systems have been used in pharmaceutical formulations to increase the apparent water solubility of poorly soluble drugs so as to improve their bioavailability. In addition, CDs and its derivatives serve to provide: (1) enhanced drug stability (thermal, photosensitivity, and chemical), (2) reduced drug mucosal irritation, (3) reduced drug resistance, and (4) controlled release of the drug [17,18]. Several synthetic strategies have been employed to engineer CD-based carriers with enhanced pharmaceutical properties along with reduced systemic toxicity [18]. For example, the introduction of bulky derivatives can limit the formation of intramolecular hydrogen bonding, consequently enhancing the aqueous solubility of CDs while improving their inclusion capacity [14]. Luke et al. reported a 35-fold increase in aqueous solubility for a sulfobutyl-ether-β-cyclodextrin (SEB-β-CD) derivative relative to native βCD [19]. In addition to chemical derivatization, CDs have been chemically conjugated to a variety of functional moieties (i.e., polymers, lipids, and peptides) to create biofunctional and supramolecular complexes [20,21]. For instance, several amphiphilic moieties have been conjugated to CDs to create self-assembling supramolecular structures with improved drug loading capacity and enhanced cellular uptake [22,23,24,25].

In the present work, we evaluate a novel βCD-based carrier modified with an unsaturated 18-carbon chain gemini surfactant conjugate, herein referred to as 18:1βCDg (Figure 1d), as a potential advanced drug delivery system for Mel in melanoma therapy. The objectives of the current study are generally two-fold: 1) to synthetically engineer a novel CD-based carrier for Mel with improved therapeutic efficiency and low carrier-cellular toxicity, and 2) to characterize the structure of the host–guest interaction between the carrier and drug. The host–guest complex of the 18:1βCDg-Mel system was investigated using 1D/2D ^1^H NMR spectroscopy in aqueous solution. NMR results herein show the formation of well-defined carrier–drug inclusion complexes. We previously reported a cationic gemini surfactant-βCD conjugate with 12-carbon chain (12 βCDg) for the delivery of a poorly soluble drugs including curcumin analogs [26,27,28] and Mel in a melanoma cell line model [29]. The 12 βCDg-Mel complex significantly improved the efficiency of Mel drug and showed no intrinsic toxicity as it did not alter the cellular death triggered by Mel [29]. However, the stability of the formed host–drug inclusion complex and the efficiency of the produced 12 βCDg-Mel system were limited due to the self-inclusion/self-assembly of the terminal alkyl chains of the carrier agent within the βCD cavity [27,28,29]. Therefore, the newly developed delivery system herein using a cationic gemini surfactant-βCD conjugate with 18-carbon tail (18:1βCDg) is anticipated to overcome these limitations. The in vitro efficiency of the 18:1βCDg-Mel complex using monolayer, 3D spheroid, and Mel-resistant melanoma cell lines demonstrates the potential applicability of the 18:1βCDg delivery system as a safe and efficient carrier for poorly soluble chemotherapeutic in melanoma therapy. This study is anticipated to provide a greater understanding of the structure–function relationship of 18:1βCDg as a carrier agent for poorly soluble drug with optimal therapeutic properties.

## 2. Material and Methods

### 2.1. Preparation of Inclusion Complexes

Melphalan (Mel) and β-Cyclodextrin (βCD) were purchased from Sigma-Aldrich (Oakville, ON, Canada). Synthesis and characterization of 18:1-7NβCD-18:1 gemini surfactant [18:1βCDg] was described elsewhere [26,30]. Figure 1 shows the chemical structures of Mel, βCD, and 18:1βCDg. For physicochemical characterization, the complexes of Mel with βCD or 18:1βCDg were prepared in different carrier-to-drug molar ratios (1:1, 2:1, 3:1, and 5:1). A stock solution of Mel (1 mg/mL) was prepared in acidified ethanol (0.1% HCl). Stock solutions of βCD and 18:1βCDg were prepared in Milli-Q water at 10 mM concentration. An appropriate volume of Mel solution was mixed with βCD and an aqueous 18:1βCDg dispersion to yield the required carrier-to-drug molar ratios. Formulations were frozen at −80 °C for 2 h and transferred to a cascade freeze dryer (Labconco, Kansas City, MO, USA) at −80 °C and 0.03 mBar vacuum and lyophilized for 24 h. The lyophilized formulations were rehydrated in water (or in deuterium oxide for ^1^H NMR and 1D/2D ROESY experiments) and shaken in the orbital shaker for 1 h at room temperature prior to evaluation.

### 2.2. Physicochemical Characterization

#### 2.2.1. Size and Zeta Potential Measurements

Eight hundred microliters of rehydrated formulations were transferred into a special cuvette (DTS1061, Malvern Instruments, Worcestershire, UK) for size distribution and zeta potential measurements using a Zetasizer Nano ZS instrument (Malvern Instruments, Worcestershire, UK). Each sample was measured in triplicate and the results are expressed as an average ± standard deviation (SD), where *n* = 3 with a corresponding polydispersity index (PDI) value.

#### 2.2.2. NMR Spectroscopy

1D/2D ^1^H ROESY NMR spectra in solution were recorded on a 500 MHz 3-channel Bruker Avance spectrometer in D_2_O at 298 K. Chemical shifts (*δ*) are reported in ppm with respect to trimethylsilane (TMS; *δ* 0.0 ppm) as external standard. ^1^H 1D spectra were obtained with water solvent suppression, a 2s recycle delay, and a 90° pulse length (10 µs). 2D ROESY NMR spectra were obtained at variable parameters which were optimized as follows: spin-lock time of 350 ms, recycle delay of 3 s with 8 scans and 1k data points. Complexation-induced chemical shift (CIS) values were calculated as Δ*δ* = *δ*_free_ − *δ*_complex_.

### 2.3. In Vitro Evaluation

Human malignant melanoma (A375) cell line (ATCC^®^ CRL-1619™, Cedarlane, Burlington, ON, Canada) was cultured in Dulbecco’s modified Eagle’s medium (DMEM) supplemented with 10% fetal bovine serum and 1% antibiotic and incubated at 37 °C under an atmosphere of 5% CO_2_/95% air. For all experiments, culturing conditions and passage numbers were kept constant.

#### 2.3.1. Determination of Half Maximal Inhibitory Concentration (IC_50_) in Monolayer Melanoma Cell Culture

A375 cell lines were seeded at a density of 1 × 10^4^ cells/well and incubated for 24 h. Cells were treated with serial concentrations of Mel (32 nM to 250 µM), either alone or as 18:1βCDg-Mel complexes at a 2:1 molar ratio, and 18:1βCDg alone (64 nM to 500 µM) in quadruplicate. After 24 h of treatment, cell viability was assessed using MTT (3-(4,5-dimethylthiazolyl-2)-2,5-diphenyltetrazolium bromide) (Sigma-Aldrich, Markham, ON, Canada) assay. The supplemented DMEM containing the treatment was removed from the wells and replaced with 0.5 mg/mL MTT in supplemented media and incubated at 37 °C for 3 h. The supernatant was removed and each well washed with phosphate-buffered saline (PBS). The formed formazan was dissolved in DMSO. Plates were incubated for 10 min at 37 °C. Absorbance was measured at 580 nm using BioTek microplate reader (Bio-Tek Instruments, Winooski, VT, USA). The half maximal inhibitory concentration (IC_50_) was determined by calculating the fraction of dead cells and plotting the data with a 4-parameter curve generated by GEN5 software from BioTek.

#### 2.3.2. In Vitro Efficiency in Spheroid Melanoma Cell

To evaluate the efficiency of developed formulations in three-dimensional cell culture, melanoma cells (A375) were cultured at a density of 1 × 10^4^ cells/well in 96-well spheroid microplate (Corning Inc., Tewksbury, MA, USA). Cells were incubated at 37 °C under an atmosphere of 5% CO_2_/95% air for 48 h prior to treatment with Mel and 18:1βCDg-Mel complexes. After 48 h, cells were treated twice (2nd treatment 24 h after the first) with Mel alone and with 18:1βCDg-Mel complexes at 2:1 molar ratio with final Mel concentrations of 30 and 80 µM in quadruplicate. The CytoTox-ONE Homogeneous Membrane Integrity Assay (Promega Corporation, Madison, WI, USA) was used to determine the lactate dehydrogenase (LDH) activity 12 h after the 2nd treatment. An equal volume of CytoTox-ONE™ Reagent (100 µL) was added to cell culture medium in each well and incubated at 23 °C for 10 min. Fifty microliters of stop solution was added to each well and fluorescence was measured using an excitation wavelength of 560 nm and an emission wavelength of 590 nm by using a BioTek microplate reader. Maximum LDH release was used as a control by adding 2 µL of lysis solution to 4 wells of nontreated cells. % cell toxicity was calculated as follows:

$$\%\;\mathrm{Cell}\;\mathrm{Toxicity}\;=\;\frac{\left(\mathrm{Experimental}\;-\;\mathrm{Culture}\;\mathrm{Medium}\;\mathrm{Background}\right)}{\left(\mathrm{Maximum}\;\mathrm{LDH}\;\mathrm{Release}\;-\;\mathrm{Culture}\;\mathrm{Medium}\;\mathrm{Background}\right)}$$

#### 2.3.3. In Vitro Efficiency in Mel-Resistant Melanoma Cell Lines

Mel-resistant melanoma cultures were created by using A375 cell line, as described previously [29]. In brief, A375 cells cultured in 25 cm^2^ tissue culture flasks and treated with increasing concentrations of Mel from 100 nM to 60 µM over 9 weeks to induce drug resistance. The Mel-resistant cells were seeded at a density of 1.5 × 10^4^ cells/well in 96-well plate and incubated for 24 h. After the incubation period, cells were treated with Mel alone and with 18:1βCDg-Mel complexes at 2:1 molar ratio with final Mel concentrations of 30 and 80 µM in quadruplicate. The MTT assay, as described above, was used to determine % cell toxicity (compared to nontreated cells).

### 2.4. Statistical Analysis

Statistical analyses were performed using SPSS software (v 24.0). Independent *t*-test and one-way analysis of variance (Bonferroni’s post hoc tests) were used. Significant differences were considered at *P* < 0.05 level.

## 3. Results and Discussion

### 3.1. Physicochemical Characterization

#### 3.1.1. H NMR and 1D/2D ROESY

Several spectroscopy methods (NMR, circular dichroism, and FT-IR) have been utilized to characterize the structure of host–guest inclusion complexes, along with *X*-ray diffractometry (XRD) and mass spectrometry (MS) [31,32]. Solution state NMR spectroscopy is a powerful tool for elucidating the molecular level structure and host/guest stoichiometry by analyzing the complexation-induced shifts (CIS). In particular, two-dimensional NMR affords an understanding of the *through-space* dipolar interactions and inclusion geometry of the guest within the βCD cavity [27,33,34]. In 2D ROESY NMR, the nuclear Överhauser effect (NOE also ROE) is employed to elucidate the noncovalent interactions between nuclei that reside in close spatial proximity (~5 Å) [35,36]. Although 2D NMR ROESY cross-correlations are related to NOE, other correlations may arise due to chemical and conformational (rotational) exchanges [37].

**Figure 2:**
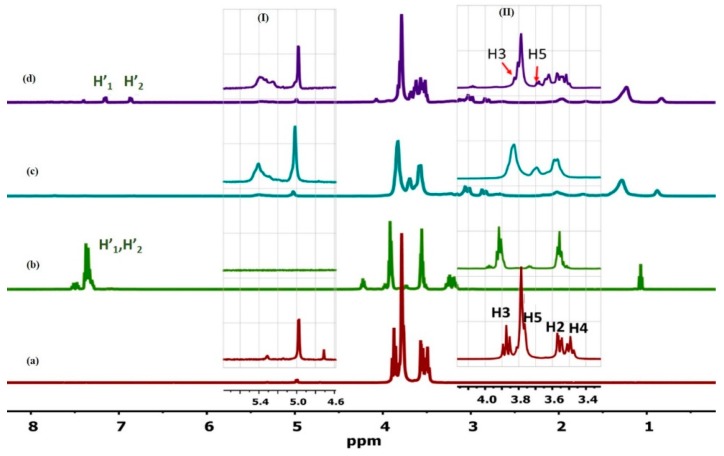
^1^H NMR spectra of (**a**) βCD, (**b**) Mel, (**c**) 18:1βCDg, and (**d**) 18:1βCDg-Mel complex at 2:1 molar ratio obtained in D_2_O at 298 K. The insets show enlarged resonance regions for the alkenic signal (H11) of the gemini (**I**) and βCD signals (**II**). Some signals for the drug (H’_1_, H’_2_) and the interior and exterior protons of βCD are labeled.

In the current work, 1D/2D ^1^H ROESY NMR was employed to elucidate the structure of the 18:1βCDg and its complexes with Mel. The ^1^H signals in Figure 2 were assigned according to previous reports [27,38] and the simulated spectra (cf. Figures S1 and S2 Supporting information). Starting with the 18:1βCDg carrier molecule, evidence of the formation of the gemini surfactant-grafted βCD is shown by the substantial broadening of the βCD resonance lines (~3.0–4.5 ppm) relative to the native βCD (Figure 2a,c, respectively), along with the emergence of the gemini surfactant signals at *δ* ~0.5–1.0 ppm (Figure 2c). Similar line broadening effects were reported previously for grafted βCD-based hosts in D_2_O [37]. The downfield shifts (Δ*δ* ~0.04–0.10 ppm, Table 1) of the external and framework protons of the 18:1βCDg (i.e., H_1_, H_4_, and H_6_; cf. Figure 1b) indicate an induced conformational change of the βCD macrocycle upon grafting. The internal cavity protons H_3_ and H_5_ of the βCD moiety of the 18:1βCDg carrier (Figure 2 highlighted area II) are characterized by upfield shifts (~−0.03 and −0.08 ppm; cf. Table 1) that indicate a possible inter- or intramolecular inclusion of part of the gemini surfactant moiety (cf. Figure 2c), consistent with shielding effects [38]. The alkenic signals of the gemini surfactant ~5.4 ppm (Figure 2 highlighted area I) are characterized by substantial broadening and upfield shift indicating a change of conformation and/or environment upon drug complexation as described above.

In the case of the 18:1βCDg-Mel, various host:guest complexes (1:1, 2:1, 3:1 and 5:1) were studied. The 1:1 and 2:1 18:1βCDg-Mel complexes showed the most affected CIS values as listed in Table 2, indicating that the 18:1βCDg carrier forms 1:1/2:1 stoichiometry with the drug. The 1D ^1^H NMR spectrum of the 18:1βCDg-Mel complex at the 2:1 mole ratio is represented in Figure 2d and reveals substantial shielding of the βCD internal protons (~3.7–4.0 ppm) and the gemini surfactant (~0.5–1.0 ppm) resonances. This provides evidence for the inclusion of the Mel within the cavity of the βCD moiety with possible involvement of the gemini surfactant moiety.

**Figure 3:**
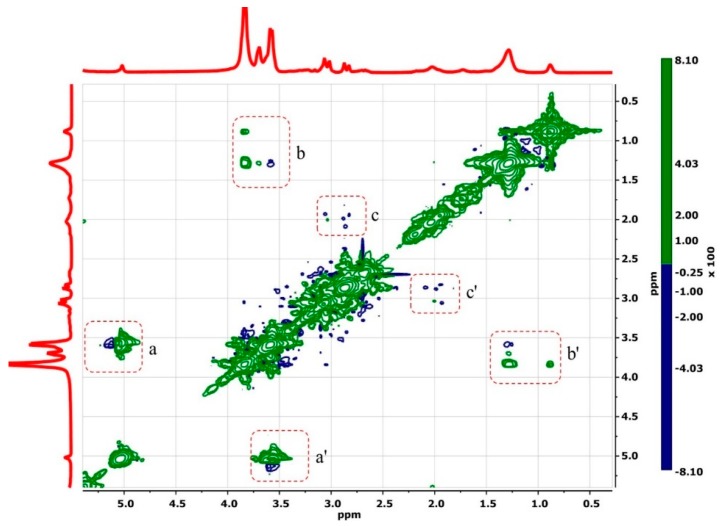
2D ROESY ^1^H NMR spectra of unbound 18:1βCDg in D_2_O and 295 K. The inter- and intra-molecular cross-peaks are shown as highlighted areas **a**–**c**, and the bar to the right shows positive and negative correlations.

**Figure 4:**
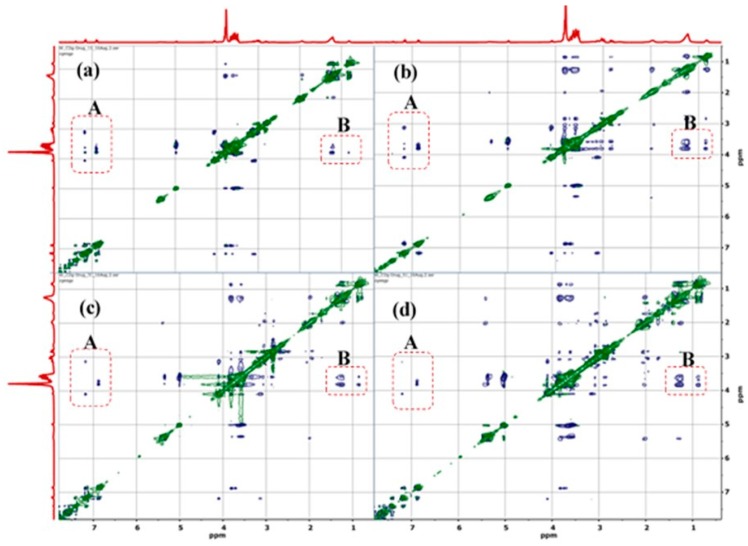
2D ROESY ^1^H NMR of the 18:1βCDg-Mel complexes at (**a**) 1:1, (**b**) 2:1, (**c**) 3:1, and (**d**) 5:1 mole ratios. The cross-peaks for the βCD-Mel and βCD-gemini surfactant interactions are shown in panels A and B. (**A**) interaction between Mel and βCD cavity and (**B**) gemini surfactant alkenic region with βCD cavity.

**Figure 5:**
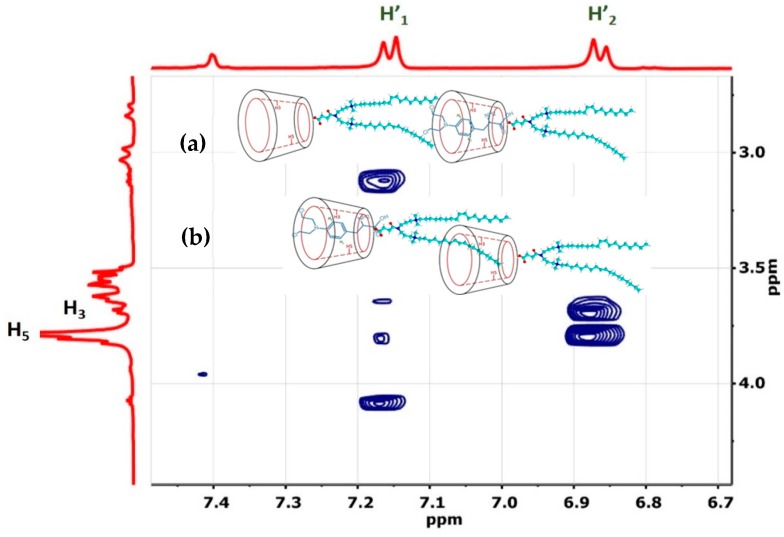
Expanded 2D ROESY ^1^H NMR of the 18:1βCDg-Mel complex at 2:1 molar ratio in D_2_O at 295 K. The possible geometries are depicted in the insets.

The 2D ^1^H ROESY NMR spectra of 18:1βCDg and its complexes with Mel are shown in Figure 3, Figure 4 and Figure 5. In Figure 3, the spectra of the unbound 18:1βCDg carrier displayed cross-peaks due to typical interactions between the backbone H_1_ proton of βCD with the cavity interior (panel a). Furthermore, various cross-peaks are arising from intra-/intermolecular interactions of the 18:1βCDg carrier (Figure 3, panel c). More recently, the self-inclusion of a 12-carbon chain gemini (12 βCDg) within the βCD cavity was reported [27]. In Figure 3, cross-peaks between the βCD cavity and the alkyl chain of the gemini surfactant (highlighted area b) are relatively weak and characterized by positive correlations (green contours in Figure 3) due to possible conformation (rotational) and chemical exchanges [37]. The formation of host–guest inclusion complexes is expected to be thermodynamically more favorable for a saturated long hydrocarbon chain than shorter ones [39]. However, intermolecular aggregation of the gemini surfactant chain is anticipated to be more prominent in the case of 18:1βCDg due to the presence of the unsaturated bond that causes the tail to bend with less flexibility, which can reduce the self-inclusion of the tail region within the βCD cavity.

**Scheme 1:**
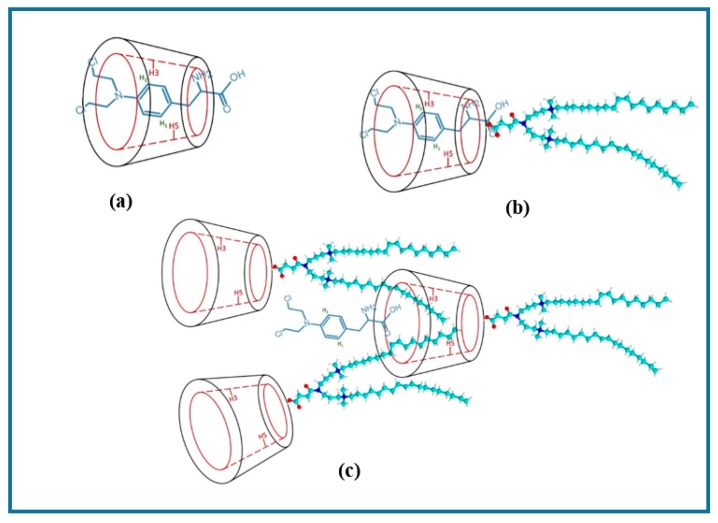
Schematic presentation of the possible inclusion geometry of the Mel and gemini surfactant; (**a**) 1:1 βCD-Mel, (**b**) 18:1βCDg-Mel at 1:1 molar ratio, and (**c**) 18:1βCD-Mel at 3:1 molar ratio. Note that other possible geometries are possible and also the structures are not drawn to scale.

The relative comparison of the 2D ROESY spectra of the complexes of 18:1βCDg with Mel at various carrier:drug mole ratios (1:1, 2:1, 3:1, and 5:1) were used to further support the effect of stoichiometry on maximum solubility (Figure 4). According to the 2D ROESY spectra in Figure 4, the increased carrier–drug interactions at the 1:1 and 2:1 mole ratios are evidenced by the intense cross-peaks and suggest optimal solubilizing conditions for the drug, more so at the 2:1 mole ratio due to the much greater intensity. It is noteworthy that the interaction between the gemini surfactant and the βCD moiety of the carrier in the presence of the Mel drug as a co-guest illustrates the importance of “cooperativity” in the host–guest inclusion complex. Evidence of cooperative association was reported recently for other βCD-based complexes [37,40]. In the present study, the “cooperative interaction” only occurred when Mel was added to the 18:1βCDg. The foregoing solubility results inferred from the 2D NMR results agree with the CIS data herein (Table 1) and with solubility evaluation results reported previously using mass spectrometry [41]. The ^1^H NMR CIS data in Table 1 provide evidence of destabilization of the carrier–drug inclusion complex due to hydrophobic effects and conformational motility of the 18:1βCDg at higher carrier loading, as indicated by the downfield shifts of the βCD extracavity (or framework) nuclei (i.e., H_1_, H_6_, and H_4_). The efficiency of 18:1βCDg to increase the aqueous solubility of Mel was evaluated using mass spectrometry, where the highest aqueous solubility was determined at the 2:1 host–guest mole ratio [41]. Increasing the 18:1βCDg-Mel mole ratios to 3:1 and 5:1 caused significant reduction in Mel solubility, consistent with the attenuated βCD-Mel signals at the expense of the βCD-gemini surfactant signals for these systems (cf. Figure 4c,d). The foregoing discussion suggests increased interaction of the gemini surfactant moiety with the βCD cavity at higher host ratio, whereas an alternate geometry involves one 18:1βCDg unit interacting with another 18:1βCDg unit inter-molecularly in a rotaxane-like fashion (cf. Scheme 1). The growing interactions between the gemini surfactant moiety at the alkenic region (~5.4 ppm; Figure 4) with the βCD interior/exterior protons at the higher host ratios indicates a possible intermolecular aggregation of the gemini surfactant around the βCD and further support the rotaxane-like structure depicted in Scheme 1. These proposed structures are further supported by the shielding and deshielding CIS trends of the external and internal βCD resonances, respectively (cf. Table 1). It is noteworthy to mention that the increased gemini surfactant-βCD interaction at the 3:1 and 5:1 mole ratios of 18:1βCDg-Mel correlate with the limited solubility of the drug at these conditions (Scheme 1c).

The 2D NMR results for the 2:1 18:1βCDg-Mel complex are shown as an expanded plot in Figure 5 and reveals NOE correlations due to interactions between the internal cavity of the βCD moiety (H_3_, H_5_) and the aromatic moiety of the drug at the H’_1_ and H’_2_ positions (cf. Figure 1a and Figure 5). These results provide unequivocal evidence for the formation of the 18:1βCDg-Mel complexes with well-defined binding and geometry that are consistent with the 1D ^1^H CIS data. The more intense cross-peaks at H5 compared to H3 in Figure 5 suggests that Mel is directionally encapsulated within the βCD cavity of the 18:1βCDg carrier, as depicted in Figure 5 (see insets). Similar geometry was deduced from the inclusion complex of Mel with pure βCD according to 2D ROESY NMR results of the βCD-Mel complex (cf. Figure S3).

Higuchi and Connors described an analytical approach to study the CD/drug solubility relation known as the phase-solubility method (Figure S4) [42]. This method examines the effect of a solubilizer (CD or ligand) on the drug being solubilized (substrate). The phase-solubility relationship describes only the solubilizing effect of the CD on the drug molecule but not the actual formation of inclusion complexes. Based on the phase-solubility diagram of Higuchi and Connors, the solubility evaluation results reported previously using flow-injection mass spectrometry of 18:1βCDg-Mel [41] and the NMR results discussed herein, we propose that the relationship between the solubility of Mel and the concentration of the host (18:1βCDg) follows either A_N_ or A-B_S_-type model. A_N_-type model indicates the formation of a host–drug soluble complex with negatively deviating isotherms, whereas the A-B_S_-type indicates the formation of a complex with limited solubility [43]. βCD-drug complexes usually follow a B-type model as a result of the limited aqueous solubility of βCD (1.85 g/100 mL). On the other hand, 18:1βCDg exhibits behavior characteristic of both βCD and cationic gemini surfactant (amorphous molecule). The solubilization effect of 18:1βCDg is combination of formation of host–guest inclusion complex and the association complex formation that involve moieties that lie in the extracavity region of the 18:1βCDg (i.e., the gemini alkyl chain) as shown by the 2D NMR results for the 2:1 18:1βCDg-Mel (Figure 4 and Figure 5).

#### 3.1.2. Size and Zeta Potential

Size of the carrier–drug complex can determine their route of administration and can affect stability, cellular uptake, biodistribution, toxicity, and clearance pathway [44,45,46]. For instance, optimal endocytosis requires particle size of the nanoparticles to be within the range of 100–200 nm [47]. In the current work, the particle size was measured for the 2:1 18:1βCDg-Mel complex with size of approximately 160 nm (Table 2). Similarly, the 18:1βCDg carrier possesses an average size of ca. 170 nm characteristic of nanoparticles. At identical concentration conditions, βCD-Mel complex and unbound βCD failed to form particles with measurable particle sizes (Table 2). In a previous report, much greater particle size was measured for a 2:1 12 βCDg:Mel complex (ca. 225 nm) in the presence of 0.5% methylcellulose as a suspending agent [29]. In the current work, the reported particle size of 18:1βCDg-Mel complex was measured in an aqueous medium without the need of a suspending agent and no visible precipitation was observed even after 24 h. It is hypothesized that the replacement of saturated 12-carbon tails with unsaturated 18-carbon alkyl tails in the case of 18:1βCDg results in the formation of a stable inclusion complex.

Aside from particle size, the surface charge of a system can affect the physiochemical stability and biological behavior of carrier-drug formulations [48]. In Table 2, the zeta potential (*ζ*) data reveals much larger positive charge (*ζ* = 46 mV) for the 18:1βCDg-Mel complex compared to the uncomplexed 18:1βCDg carrier (*ζ* = 14 mV). This effect can be explained by the difference in the measured pH values between the complex (pH 2.8) and the 18:1βCDg aqueous dispersion (pH 4.7). Low acidic pH can lead to the protonation of melphalan, causing more positive *ζ*-values [49].

### 3.2. In Vitro Evaluation

The in vitro activity of 18:1βCDg-Mel complex, optimized at the 2:1 mole ratio, was evaluated in different melanoma cell line models. The in vitro studies were performed to evaluate the ability of the carrier to: (1) enhance the efficiency of Mel in standard monolayer cell lines; (2) enhance the penetration of the Mel in 3D melanoma tissue culture; (3) overcome drug resistance in Mel-resistant cells.

A standard monolayer A375 cell line was used to determine the IC_50_ of Mel (cf. Table 3, Figure S5). The concentration of Mel required to induce cell death in the presence of 18:1βCDg carrier at the 2:1 (18:1βCDg-Mel) molar ratio was significantly lower with IC_50_ values of 27 ± 1 µM compared to Mel- alone with IC_50_ = 98 ± 1 µM (*P* < 0.05). Additionally, the IC_50_ value for the unbound carrier 18:1βCDg was ~89 µM. These results indicate that the 18:1βCDg-Mel complex can significantly improve the efficiency of Mel with low carrier-specific toxicity of the cells. Recently, we reported that βCD-gemini system did not alter the cell death pathway induced by Mel, as shown by apoptosis and cell cycle analyses, indicating that the βCD-modified gemini surfactant bears no intrinsic toxicity to the cells [29].

**Figure 6:**
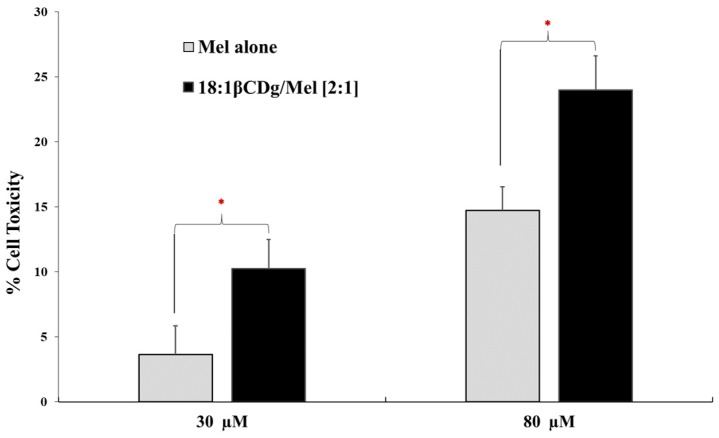
Cytotoxic efficiency of the Mel-alone and 18:1βCDg-Mel complexes (at 2:1 molar ratio) in 3D spheroid A375 melanoma cells. A375 cells were seeded at 1 × 10^4^ cells/well in Corning^®^ spheroid 96-well microplates and treated twice (24 h and 36 h after seeding) with Mel-alone and 18:1βCDg-Mel complexes with final Mel concentrations of 30 µM and 80 µM. Cell toxicity was reported as lactate dehydrogenase (LDH) activity. *N* = 3 ± SD * indicates significance (*P* < 0.05).

To mimic the complex three-dimensional (3D) architecture of a solid tumor and to investigate the cell penetration ability of the βCD-modified gemini surfactant and hence the improvement in the drug permeability, a spheroid melanoma cell culture was created. Treatment of spheroid melanoma cell culture with 30 µM 18:1βCDg-Mel complexes at Mel final concentration of 30 µM (the approximate IC_50_ value in monolayer culture) caused 10% cell death, a three-fold increase in cell toxicity compared to Mel alone (cf. Figure 6). These results are based on the differences in IC_50_ values for Mel (98 µM) and 18:1βCDg-Mel (27 µM) in monolayer A375 cell lines (cf. Table 3). Similarly, treatment of spheroid melanoma cell culture at greater Mel final concentration of 80 µM (recapitulating the IC_50_ value of naked Mel in monolayer) showed 24% cell toxicity with 18:1βCDg-Mel complexes, compared to 14% for Mel-alone at the same conditions (Figure 6). While cell toxicity for 18:1βCDg-Mel complexes (at both 30 and 80 µM) are lower than cell death in A375 monolayer cell lines, the results are encouraging for future formulation development upon accounting for the higher complexity of the spheroid cell culture and smaller direct contact area with the drug dispersed in the cell culture medium compared to monolayer cultures. Nevertheless, formulating Mel with 18:1βCDg caused a significant increase in the cytotoxicity of the drug at both concentrations. In our previous work, 12 βCDg did not improve the delivery of Mel in the tumor spheroids of A375 cells as no such difference were found between the IC_50_ values between Mel-alone and the Mel-12 βCDg formulation [29]. Thus, we hypothesize that the 18:1βCDg, with unsaturated 18:1 tail, forms more favorable inclusion complexes with Mel than the 12 βCDg with 12-carbon alkyl tail. This may be attributed to the combined effect of a more prominent inclusion binding along with secondary interactions due to association of the non-included C18:1 gemini chain that resides in the interstitial region of 18:1βCDg, as described in the NMR results (Scheme 1).

**Figure 7:**
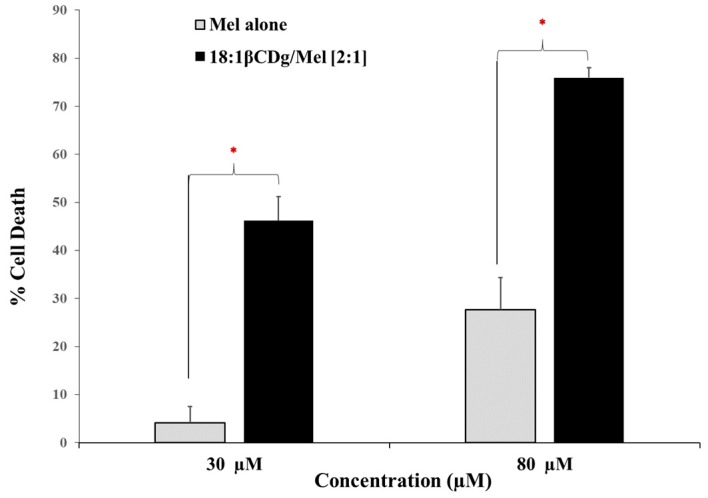
Cytotoxic efficiency of the Mel-alone and 18:1βCDg-Mel complexes (at 2:1 molar ratio) in Mel-resistant A375 melanoma cells. A375 cells were seeded 1.5 × 104 cells/well in 96-well plate and after 24 h treated with Mel-alone and 18:1βCDg-Mel complexes with final Mel concentrations of 30 µM and 80 µM. Cell death was reported as using MTT assay in comparison with nontreated cells. *N* = 3 ± SD * indicates significant (*P* < 0.05).

Advanced drug delivery systems can provide a potential avenue to overcome drug resistance by enhancing the bioavailability of the drug target at the tumor site. This can be achieved by shielding the drug molecule via complex formation to minimize its efflux from the cell [50]. Previously, we reported that formulating Mel with 12 βCDg significantly improved the efficiency of the drug in Mel-resistant A375, whereas Mel alone showed minimum cell death at the same concentration, in line with the anticipated effect [29]. In the current work, we evaluated the capability of newly designed βCD-modified gemini surfactant (18:1βCDg) to enhance the cytotoxic action of melphalan in Mel-resistant cultures (Figure 7). The Mel-resistant A375 cells showed low cell toxicity toward melphalan even at the highest concentration that was evaluated here (500 µM of melphalan). Therefore, we used 30 and 80 µM melphalan based on the known IC_50_ values of melphalan and the corresponding IC_50_ values of 18:1βCDg alone in a standard monolayer cell culture assay (Table 3, Figure S5). Treating the Mel-resistant A375 melanoma cells with Mel-alone at 30 and 80 µM caused low cell death (4% and 27%, respectively). However, after treating the Mel-resistant cells with 18:1βCDg-Mel complexes at final Mel concentration of 30 and 80 µM, a significant recovery of the activity was observed (46 and 76% cell death, respectively), as shown in Figure 7. These results suggest that the 18:1βCDg delivery system was able to overcome the apparent drug resistance and enhance the treatment efficacy.

## 4. Conclusions

In this work, a novel carrier based on derivatization of βCD with an unsaturated 18-carbon chain gemini surfactant conjugate (18:1βCDg) was characterized and its potential as an advanced drug delivery system for Melphalan (Mel) drug in melanoma therapy was evaluated. The 18:1βCDg carrier and its complexes with Mel drug were characterized using 1D/2D NMR spectroscopy, along with the measurement of particle and zeta potential in aqueous solution. The 18:1βCDg carrier improves the solubility of Mel through formation of favorable inclusion complexes at the 2:1 mole ratio, as supported by 1D CIS data and 2D ROESY NMR results. The inclusion of Mel involves a well-defined geometry where the drug is directionally encapsulated within the internal apolar cavity of the βCD carrier system, according to 2D ROESY NMR results. The self-inclusion of the terminal part of the gemini alkyl chain within the βCD cavity cannot be ruled out especially at equimolar carrier/drug ratios. However, these effects are minimized at carrier mole ratios >1:1 due to hydrophobic aggregation of the carrier chains. The measured particle sizes of the unbound 18:1βCDg carrier (ca. 170 nm) and the 2:1 carrier: drug complex (160 nm) are within nanoparticle size limits (100–200 nm). Thus, the 18:1βCDg carrier affords optimum stability, cellular uptake, biodistribution, toxicity, and clearance pathway of the reported formulation. As well, the in vitro evaluations of the optimized 18:1βCDg-Mel formulation in the presence of various melanoma models (i.e., monolayer, 3D spheroid, and Mel-resistant melanoma cells) resulted in significantly improved cytotoxic efficiency of the Mel in all cases. We are envisioning future studies to elucidate the pathways of cell penetration and of mechanism overcoming drug resistance of the 18:1βCDg-drug complexes. This knowledge will enable us to further optimize the structure that aims to improve efficiency and increase penetration ability into spheroids.

## Figures and Tables

**Table 1:** Complexation-induced chemical shift (CIS) data of the host (βCD and 18:1βCDg) and its complexes with Melphalan. The CIS values (in brackets) were calculated as ∆*δ* = *δ*_complex_ − *δ*_free_, where negative and positive values represent shielding (upfield) and deshielding (downfield) effects, respectively. * Chemical shift values are difficult to decipher.

Host:Guest	Ratio	^1^H Nuclei					
		H1	H2	H3	H4	H5	H6
**βCD**	--	4.98	3.56	3.87	3.49	3.77	3.76
βCD:Mel	2:1	4.97 (−0.01)	3.57 (0.01)	3.85 (−0.02)	3.48 (−0.01)	3.70 (−0.07)	3.78 * (0.02)
18:1βCDg	--	5.02 (0.04)	*	3.84 (−0.03)	3.59 * (0.10)	3.69 (−0.08)	3.83 (0.07)
18:1βCDg:Mel	1:1	4.97 (−0.01)	3.57 (0.01)	3.80 (−0.07)	3.50 (0.01)	3.61 (−0.16)	3.77 (0.01)
18:1βCDg:Mel	2:1	4.98 (0.00)	3.57 (0.01)	3.81 (−0.06)	3.52 (0.03)	3.62 (−0.15)	3.79 (0.03)
18:1βCDg:Mel	3:1	4.99 (0.01)	3.57 (0.01)	3.81 (−0.06)	3.52 (0.03)	3.63 (−0.14)	3.79 (0.03)
18:1βCDg:Mel	5:1	5.01 (0.03)	3.60 * (0.04)	3.83 (−0.04)	3.55 * (0.06)	3.67 (−0.10)	3.82 (0.06)

**Table 2:** Physiochemical properties (size and zeta potential) of different melphalan formulations. (--) indicates unmeasurable size or zeta potential. ***^a^*** Results are an average of n ≥ 3 ± standard deviation (SD).

Component^a^	Size nm ± SD	PDI	Zeta Potential mV ± SD
Mel (1 mM)	--	--	--
βCD (2 mM)	--	--	--
18:1βCDg (2 mM)	170 ± 17	0.329 ± 0.047	+14 ± 3
βCD-Mel (2:1 mole ratio)	--	--	--
18:1βCDg-Mel (2:1 mole ratio)	160 ± 15	0.430 ± 0.04	+46 ± 2

**Table 3:** The 50% of inhibitory concentration (IC_50_) of: [A] Mel-alone, [B] 18:1βCDg alone, and [C] 18:1βCDg-Mel complexes at 2:1 molar ratio. IC_50_ determined in A375 monolayer cell lines by using MTT (3-(4,5-dimethylthiazolyl-2)-2,5-diphenyltetrazolium bromide) assay. IC_50_ was established by calculating the fraction of dead cells and plotting the data with a 4-parameter curve. *N* = 3 ± SD.

	Treatment	IC_50_
[A]	Mel	98 ± 1 µM
[B]	18:1βCDg	89 ± 2 µM
[C]	18:1βCDg-Mel [2:1 molar ratio]	27 ± 1 µM

## Supplementary Materials

The following are available online at https://www.mdpi.com/1999-4923/11/9/427/s1, Figure S1. Predicted ^1^H NMR spectra of Melphalan. Spectra was created using nmrdb online tool (www.nmrdb.org). Figure S2. Predicted ^1^H NMR spectra of 18:1 gemini surfactant. Spectra was created using nmrdb online tool (www.nmrdb.org). Figure S3. 2D ROESY spectrum of βCD-Mel at a 2:1 host-guest mole ratio, showing cross-peaks between βCD internal 1H cavity and Mel nuclei. Figure S4. Phase solubility diagram. Figure S5. Cytotoxic efficiency of Melphalan in human malignant melanoma (A375) cell line). A375 cells were seeded at 1 × 10^4^ cells/well in 96-well plate. Toxicity was reported using MTT Assay in comparison with non-treated cells (100% viability). *N* = 3 ± SD.
