# Supplementary material for: Inclusion Complexes of Melphalan with Gemini-Conjugated β-Cyclodextrin: Physicochemical Properties and Chemotherapeutic Efficacy in In-Vitro Tumor Models
Source: Pharmaceutics. 2019 Aug 22;11(9):427. doi: 10.3390/pharmaceutics11090427 (PMC6781286; doi:10.3390/pharmaceutics11090427)
Supplement: Supplementary file 1 [file pharmaceutics-11-00427-s001.pdf]

pharmaceutics

MDPI

Supplementary Materials: Inclusion Complexes of Melphalan with Gemini-Conjugated β-Cyclodextrin: Physicochemical Properties and Chemotherapeutic Efficacy in In-Vitro Tumor Models

Waleed Mohammed-Saeid, Abdalla H Karoyo, Ronald E Verrall, Lee D Wilson and Ildiko Badea

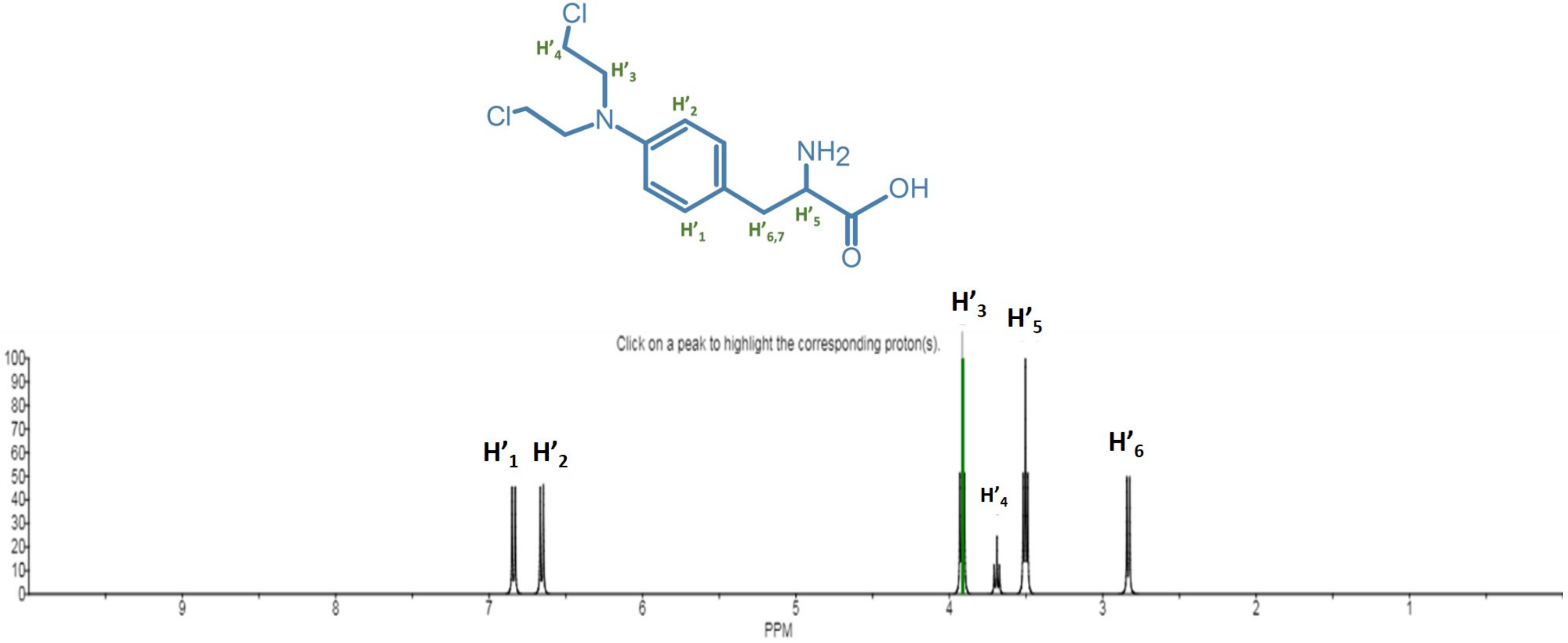

Figure S1. Predicted ^{1}H NMR spectra of Melphalan. Spectra created using nmrdb online tool (www.nmrdb.org)[1].

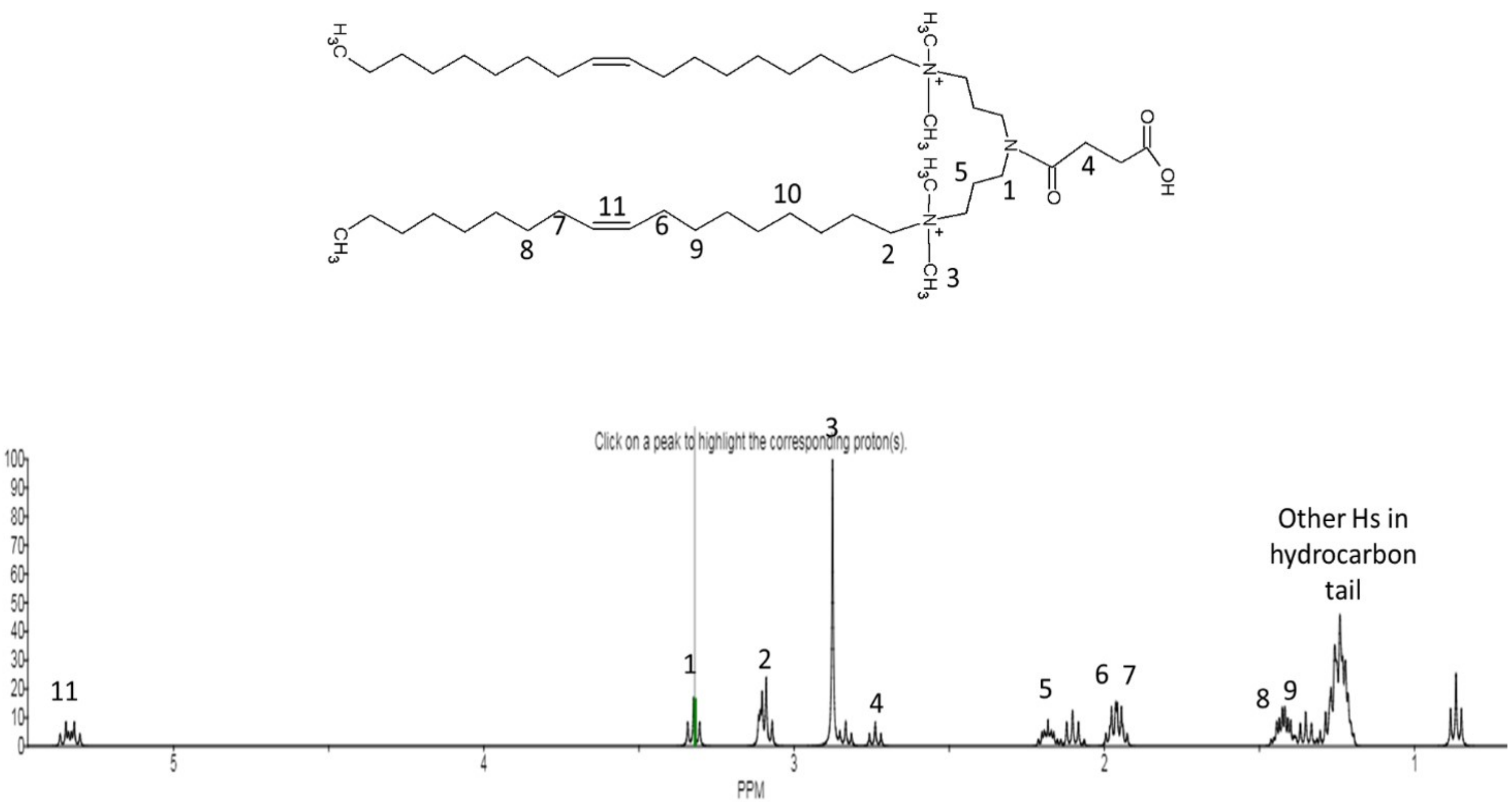

Figure S2. Predicted ^{1}H NMR spectra of 18:1 gemini surfactant. Spectra created using nmrdb online tool (www.nmrdb.org)[1].

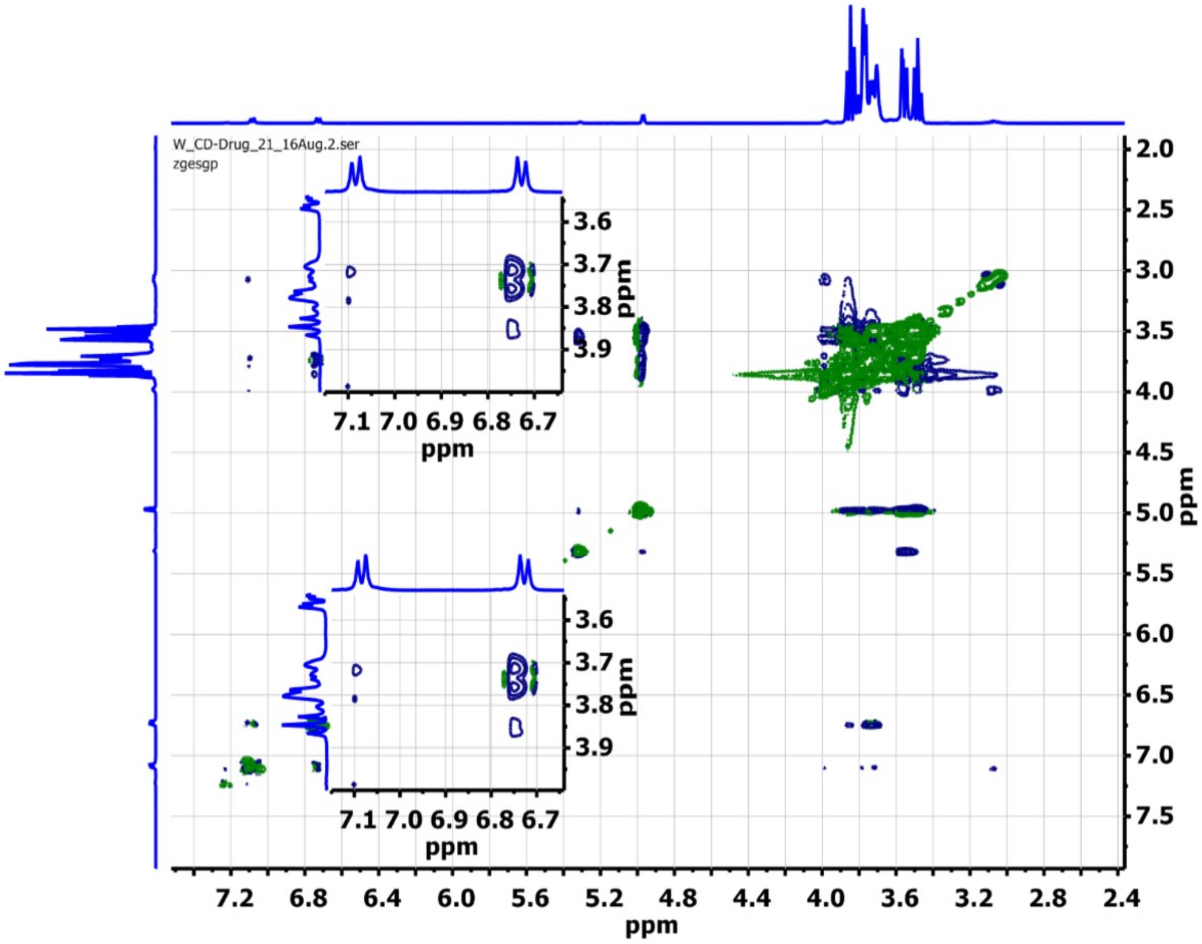

Figure S3. 2D ROESY spectrum of βCD-Mel at a 2:1 host-guest mole ratio, showing cross-peaks between βCD internal ^{1}H cavity and Mel nuclei.

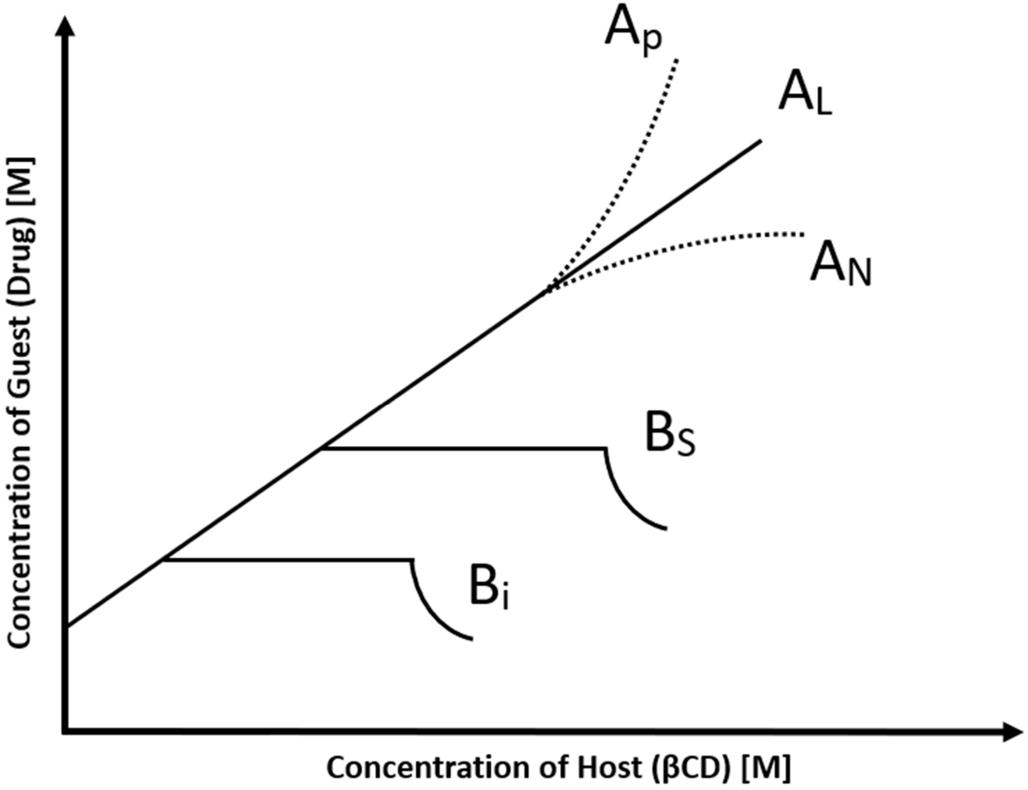

Figure S4. Phase solubility diagram[2].

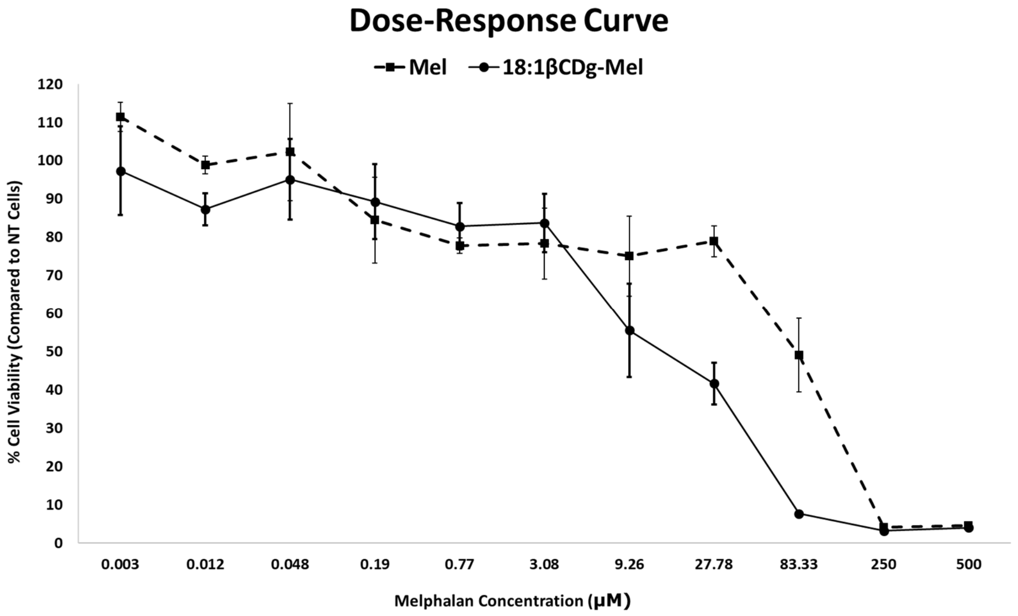

Figure S5. Cytotoxic efficiency of Melphalan in human malignant melanoma (A375) cell line). A375 cells were seeded at 1 × 10^4 cells/well in 96-well plate. Toxicity was reported using MTT Assay in comparison with non-treated cells (100% viability). $N = 3 \pm$ SD.

Reference

1. Banfi, D., Patiny, L. www. nmrdb. org: Resurrecting and Processing NMR Spectra On-line. *Chimia Int. J. Chem*. **2008**, *62*, 280–281.
2. Higuchi, T., Connors, A. Phase-solubility techniques. *Adv. Anal. Chem. Instrum.* **1965**, *4*, 117–210.
